# Supplementary material for: Electrochemical Immunodetection of Bacillus anthracis Spores
Source: Sensors (Basel). 2025 Sep 24;25(19):5948. doi: 10.3390/s25195948 (PMC12527017; doi:10.3390/s25195948)
Supplement: Supplementary file 1 [file sensors-25-05948-s001.zip › sensors-3836141-supplementary.pdf]

To enable precise and controlled surface modification of the working electrode, a dedicated electrode holder was designed and fabricated at the Military Institute of Chemistry and Radiometry. The holder features a circular top opening with a diameter corresponding to the active surface area of the electrode, allowing selective access for surface functionalization.

The electrode was securely mounted in a specially designed slot within the holder in such a way that the reference and counter electrodes were physically shielded, while maintaining unobstructed access to the electrical contacts. This design was essential, as certain modification steps were performed with the electrode actively connected to a potentiostat.

To minimize evaporation of the reaction solution and ensure stable environmental conditions during modification, the holder's opening was sealed. This setup ensured that each functionalization step was confined exclusively to the working electrode's active area, under reproducible and well-controlled conditions.

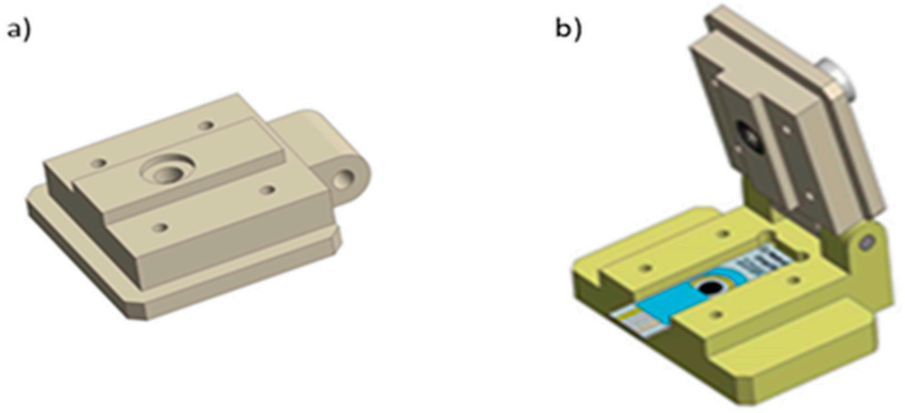

Figure S1. Holder scheme. On the left a) upper cover with reagent inlet, on the right b) complete holder with electrode inside.
